# Across-Shelf Transport of Bivalve Larvae: Can the Interface between a Coastal Current and Inshore Waters Act as an Ecological Barrier to Larval Dispersal?

**DOI:** 10.1371/journal.pone.0048960

**Published:** 2012-11-12

**Authors:** Charles E. Tilburg, Michael A. McCartney, Philip O. Yund

**Affiliations:** 1 Department of Marine Sciences, University of New England, Biddeford, Maine, United States of America; 2 Department of Biology and Marine Biology, University of North Carolina-Wilmington, Wilmington, North Carolina, United States of America; University of Vigo, Spain

## Abstract

Using an integrated physical and biological approach, we examined across-shelf advection and exchange and the associated transport of bivalve larvae in the presence of a strong coastal current separated from the coast by a stratified inshore environment. We tested the hypothesis that the interface of the coastal current and inshore waters can act as an ecological barrier to across-shelf transport of larvae but can be overcome by wind- or tidally-induced transport. Our study region in the Gulf of Maine encompasses a coastal current that diverges from the coast as it moves downshelf. The region inshore of this current is home to several species that exhibit limited recruitment in spite of extensive upshelf larval sources. Analysis of surface water temperatures and wind velocities revealed episodic decreases in temperature along the coast correlated with alongshelf (but not upwelling) winds, indicating wind-forced onshore movement of the cold coastal current. Such wind-driven onshore migrations are more common along the northern portion of the study region where the coastal current is near the coast, tidal currents are strong, and wind directions are more conducive to onshore migration, but rarer further south where the interface between inshore waters and the coastal current is further offshore and suitable wind events are less common. The distribution of bivalve larvae was consistent with the physical measurements. There was little across-shelf variation in larval abundance where the current abuts the coast, indicating strong across-shelf exchange of larvae, but strong across-shelf variation in larval density where the stratified inshore waters separate the current from the coast, indicating weak across-shelf transport of larvae. Our results suggest that the interface between the coastal current and inshore waters may constitute a major ecological barrier to larval dispersal in the southern part of the region that may only be overcome by rare, strong wind-forced events.

## Introduction

Answering questions about larval transport and population connectivity generally requires integrated physical and biological studies [1]. Such approaches have yielded considerable insight into larval transport pathways. Work to date on coastal marine organisms has made great advances in understanding the physical mechanisms responsible for alongshelf transport [2], [3], although the causes of across-shelf transport are still under debate [4]. To some extent, this focus reflects the general understanding of the physical flow field. Alongshelf velocities tend to be larger and more easily quantified than across-shelf velocities in the coastal ocean [3]. However, alongshelf transport is only one component of the connectivity equation. Long-distance dispersal of coastal organisms (intertidal and shallow subtidal) inevitably involves two across-shelf phases, as larvae are transported from the inshore waters into larger scale coastal currents, and then eventually return to the inshore water in another location. The last decade has seen significant progress on these across-shelf transport phases (mainly the onshore portion), with examinations of the roles of upwelling [5], downwelling [3], tides [6], [7] and internal waves [8], [9] on larval movement.

Buoyant coastal currents driven by river discharge are a common feature of coastal circulation and, hence, a likely mechanism for alongshelf larval transport [3], [10]. Exchange between these coastal currents and inshore waters, where larvae of coastal species are produced and must ultimately return to settle, tends to be dominated by flows driven by wind [11], [12], tides [13], and flow instabilities [14]. Although a number of studies [15]–[17] have developed statistical models that link larval settlement with processes responsible for across-shelf transport, few [18], [19] have examined the interaction of these physical mechanisms with a coastal current and the subsequent effect on across-shelf larval transport.

**Figure 1:**
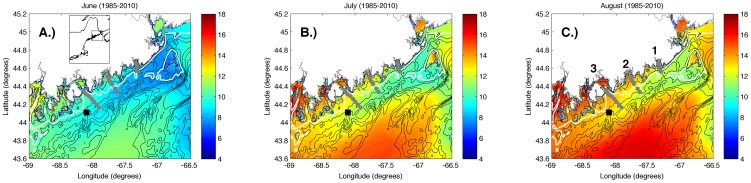
Climatological sea surface temperature (°C) of study area for (A.) June, (B.) July, and (C.) August. The gray circles indicate the locations of the CTD and larval sampling stations on the three transects. The numbers in (C.) indicate the inshore end of each transect. Transect #1 is farthest upshelf (northeast). Transect #3 is farthest downshelf (southwest). The white line represents the 75 m isobath. The black square indicates the location of buoy EB44034, which was the source of wind data.

The coastal circulation of the Gulf of Maine presents an ideal system in which to examine the effects of winds and tides on across-shelf transport of larvae in the presence of a coastal current. The northwestern portion of the Gulf of Maine (Figure 1) is characterized by a number of small estuaries, a stratified inshore region, an expanding continental shelf, and a strong coastal current (the Eastern Maine Coastal Current, or EMCC) that is located offshore. Larvae of numerous species are expected to occupy both the coastal current and the inshore waters of the region [20]–[22].

In this study, we explored across-shelf advection and exchange and the associated transport of bivalve larvae. We tested the hypothesis that the interface of a coastal current and inshore waters can act as an ecological barrier to across-shelf transport of larvae and asked whether that barrier can be overcome by strong wind-induced transport or tidal currents. As a zone of limited exchange, we view the interface between these water masses not as a physical barrier analogous to a wall, but rather as a region of reduced advection [23]. We examined the following two questions concerning the distribution of bivalve larvae along the coast of Maine: (1) Is the across-shelf structure of larval abundance consistent with physical parameters such as horizontal exchange and advection? (2) Can wind-driven flow and tidal currents transport larvae across-shelf in the presence of a coastal current?

## Materials and Methods

### Study Site

The Gulf of Maine is a marginal sea in the northwest Atlantic Ocean that covers 93,000 km^2^ and has over 12,000 km of coastline. The circulation and density structure of the Gulf of Maine are affected by heat flux from the atmosphere [24], [25], freshwater river discharge [26], tidal forcing [27], and exchange with the Atlantic Ocean [28]. The coastal circulation patterns along the western boundary of the Gulf of Maine are dominated by the Gulf of Maine Coastal Current, which consists of two branches with intermittent interaction [27]–[29]: the Western Maine Coastal Current (WMCC) and the Eastern Maine Coastal Current (EMCC). Strong tidal currents in the eastern Gulf of Maine tend to mix the waters of the EMCC, creating little vertical stratification in salinity and velocity [28]. The WMCC is fed by the combined discharge of a number of rivers along the coast and is more vertically stratified than the EMCC [30], which is driven by discharge from the St. John and St. Croix Rivers [26] and by water from the Scotian Shelf [28]. In the Gulf of Maine, freshwater emanating from the river mouths turns downshelf (southwestward) due to the Coriolis effect, supplementing the remotely-forced, geostrophic flow along the coast. Hence, the direction of both the EMCC and WMCC oppose the direction of the predominant northeastward winds in the region [21]. Transport within both coastal currents is greatest in the spring and summer, when river discharge is at a maximum [31]. The waters of the two currents can be readily distinguished from adjacent water masses by their temperature signals [28]. The inshore edge of the EMCC is bounded by the 75 m isobath, resulting in inshore waters that are warmer and more stratified than the offshore EMCC (Figure 1).

Our study site encompasses a region in which the EMCC diverges from the coast as it travels southwestward. Three across-shelf transects (gray circles in Figure 1) were selected to occupy both the stratified inshore region and the colder EMCC offshore. Transects 1, 2, and 3 (numbers in Figure 1c) originated in Machias, Pleasant, and Frenchman Bays, respectively. There is a strong across-shelf gradient in temperature due to the presence of the EMCC as well as a strong alongshelf gradient due to both warming of EMCC waters [28] as they mix with the warmer Gulf of Maine waters and the offshore migration of the EMCC to the southwest (Figure 1). The number of hydrographic and larval sampling stations on each transect increased with transect length from northeast to southwest (gray circles in Figure 1; 4 stations on transect 1, 6 on transect 2, and 8 on transect 3). Stations were approximately evenly spaced (∼3.6–4.3 km) and numbered sequentially from inshore to offshore.

### Physical Data

Hydrographic data were collected on four cruises in 2010: June 9–11, July 20–22, August 3–6, and August 24–26. During each cruise, a manually deployed Seabird SBE 19plus V2 SEACAT CTD (Conductivity-Temperature-Depth instrument) was used to determine the vertical structure of the water column at each station on the transects. Sea surface temperature measurements were made by AVHRR (Advanced Very High Resolution Radiometer) satellite, with data received and processed at the ground station at the Satellite Oceanography Laboratory at the University of Maine (pers. comm. A. Thomas). Because the hydrographic transects and satellite measurements can provide only limited snapshots of the physical flow field, moored temperature observations were collected at several inshore stations on each transect. Subsurface temperature data were collected at a depth of 5 m using Onset Instruments temperature loggers (Pendant and Tidbit V2) sampling at 10 min intervals along the three transects from June 20 to August 26, 2010. Unfortunately, extensive bottom-trawling activity on the offshore ends of the transects and the strong currents of the EMCC precluded deployment of moored temperature loggers at most stations in the EMCC. Across-shelf temperature gradients were calculated by subtracting the 5 m offshore temperatures (station 3 for transect 1 and station 4 for transect 2) from 5 m inshore temperatures and dividing by the distance between the stations. Wind data were collected from National Oceanic and Atmospheric Administration's Eastern Maine Shelf buoy (EB 44034), which is located approximately 20 km south of transect 3 (black square in Figure 1). Wind data were converted into wind stress components.

To examine the flow field, a total of 23 drifters were released during this study: 10 surface drifters and 13 drogued at a depth of 5 m. The surface drifters were similar to the commonly used ‘Davis’-style drifters [32], [33] and consisted of a low cost GPS receiver and transmitter attached to the top of a 1.3 m long ×0.05 m diameter PVC pipe connected to sails constructed of fiberglass rods and vinyl cloth. The drogued drifters consisted of a GPS receiver and transmitter attached to the top of a 1 m long ×0.1 m diameter PVC pipe connected to a 5 m ×1 m holey-sock drogue centered at a depth of 5 m. Both types of drogues comply with the World Ocean Circulation Experiment specifications of 40:1 drag ratio. To supplement our project-specific deployments, 177 additional drifter trajectories in the study region from 1998–2009 were accessed from the NOAA drifter database at http://www.nefsc.noaa.gov/epd/ocean/MainPage/ioos.html. Drifter velocities were first calculated for each trajectory and binned in 0.05° boxes. Calculations of average horizontal velocity, mean kinetic energy (MKE), and eddy kinetic energy (EKE) were then carried out using published methods [34].

When necessary, the wind and temperature data were filtered using a Lanczos low-pass filter with a cut-off frequency of 1/36 hour^−1^ to remove high-frequency variation such as wind gusts or diel and tidal motions [15]. Power spectra, which show variation in the temperature signal as a function of frequency, were calculated from the unfiltered data to examine the effects of physical mechanisms acting at different time scales. The confidence levels and intervals for correlations, coherence, and spectra were computed following published methods [35], [36]. All reported correlation coefficients were significantly different from zero at the 95% confidence level. For the moored temperature and wind data series, the degrees of freedom (*N_eff_*) replaced the number of observations (*N*) in determining significance. *N_eff_* was calculated by dividing the total time of the observations by the time of the first zero crossing of the autocorrelation function [35].

### Larval Sampling

No specific permits were required for the described field studies, since we did not work on a regulated, endangered, or protected species. On each cruise, the density of bivalve larvae was quantified at each station along each transect. An electric pump was used to filter 100 L of water through two nested plankton nets to yield a retained size fraction of 50 to 333 microns. Three replicate samples were collected at both 0.25 and 5 m depths, but bivalve larvae were vastly more abundant at depth. Consequently, only 5 m samples are formally analyzed here, although 0.25 m densities are presented for qualitative comparison. Samples were enumerated at 40× under a dissecting microscope. Sampling coincided with times during which larval mussels (mainly *Mytilus edulis* and *M. trossulus*) were abundant, although larvae of other mussel species and clams were also present. Bivalve larvae constituted the vast majority of the meroplankton community. For the purpose of this paper, we did not identify larvae to the family or genus level via inverted or electron microscopy [37]. Because the species that is most likely to serve as a tracer for across-shelf mixing (i.e., *M. trossulus*, see discussion) can only be distinguished from its close congener via genetic markers, we are currently adapting existing molecular techniques [38] to enable species-level larval identification in future publications. All of the candidate sample species produce weakly swimming larvae that are capable of some vertical movement. However, extensive vertical mixing in the EMCC [28] would appear to preclude selective tidal stream transport [39] via vertical migration in that water body. Nevertheless, vertical migration could have played a role in determining larval distributions on the inshore ends of the transects that were located in stratified water masses.

Because our bivalve larvae samples contain a mix of species, multiple sources of origin are possible from both within and outside the EMCC. A sample from any given date and station may have originated on either the inshore side of the sample domain, or upshelf, with transport into the EMCC occurring in an upshelf region where the EMCC contacts the shore. While this multi-taxon approach has limits, it yields sufficient information to evaluate the consequence of across-shelf exchange between the EMCC and inshore waters on the horizontal homogeneity of larvae. Larval bivalve density data at 5 m met ANOVA assumptions and were analyzed by testing for station effects on each transect and cruise combination via one-way ANOVA. If a significant station effect was identified, we then used Tukey-Kramer post-hoc tests to evaluate pairwise differences among the stations.

## Results

### Hydrographic Transects

**Figure 2:**
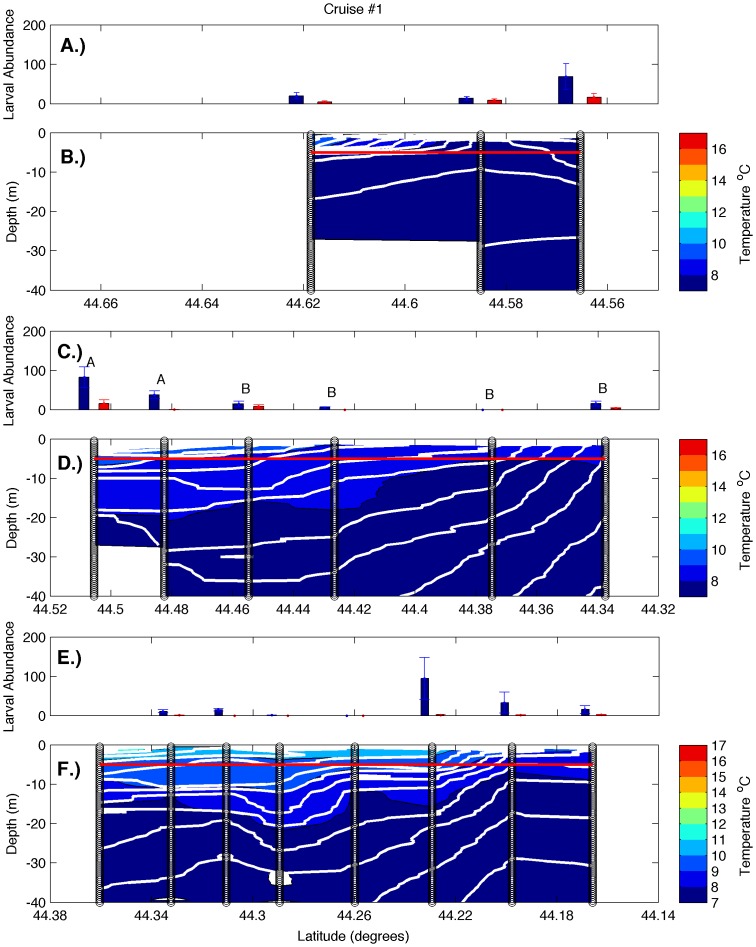
Larval abundance and water column structure on June 8–10, 2010. Panels represent larval abundance and water column structure, respectively, for transects 1 (A, B), 2 (C, D), and 3 (E, F). Larval abundance is presented as larvae m^−3^ at 5 m (blue bars) and 0.25 m (red bars). Error bars represent one standard error. The red line indicates the depth at which 5 m samples were collected. Variation in larval density among stations was only significant on transect 2 (one-way ANOVA, p<0.005), and letters indicate 5 m stations that did not differ significantly from one another (Tukey-Kramer post-hoc test, p>0.05). Vertical black lines represent the locations of CTD and larval sampling stations, color gradations represent water temperatures, and the white contour lines represent isopycnals.

**Figure 3:**
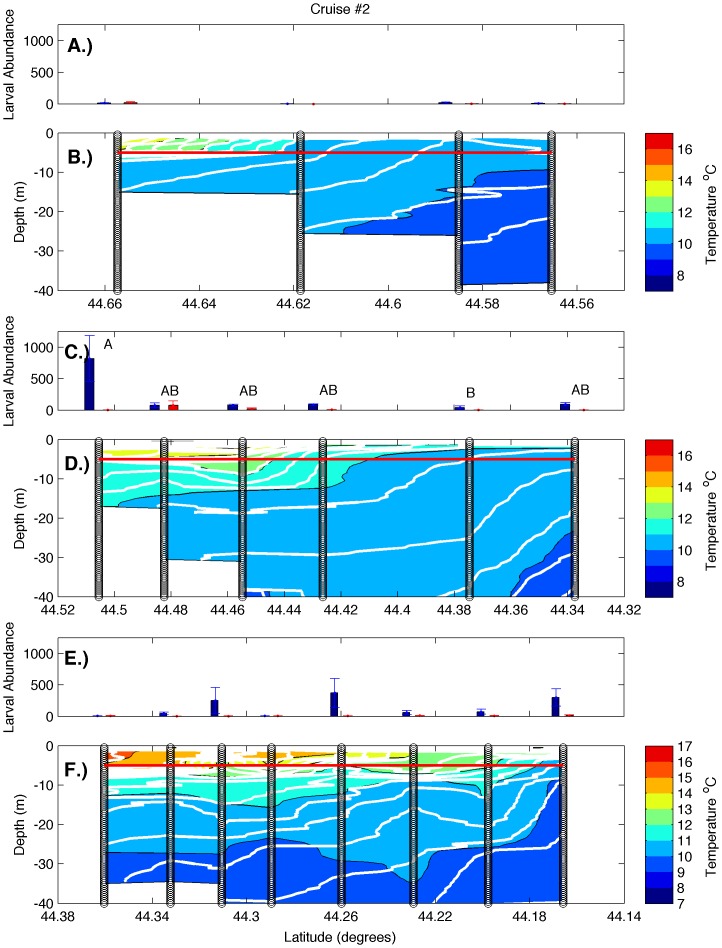
Larval abundance and water column structure on July 20–22, 2010. Panels represent larval abundance and water column structure, respectively, for transects 1 (A, B), 2 (C, D), and 3 (E, F). Larval abundance is presented as larvae m^−3^ at 5 m (blue bars) and 0.25 m (red bars). Error bars represent one standard error. The red line indicates the depth at which 5 m samples were collected. Variation in larval density among stations was only significant on transect 2 (one-way ANOVA, p<0.05), and letters indicate 5 m stations that did not differ significantly from one another (Tukey-Kramer post-hoc test, p>0.05). Vertical black lines represent the locations of CTD and larval sampling stations, color gradations represent water temperatures, and the white contour lines represent isopycnals.

**Figure 4:**
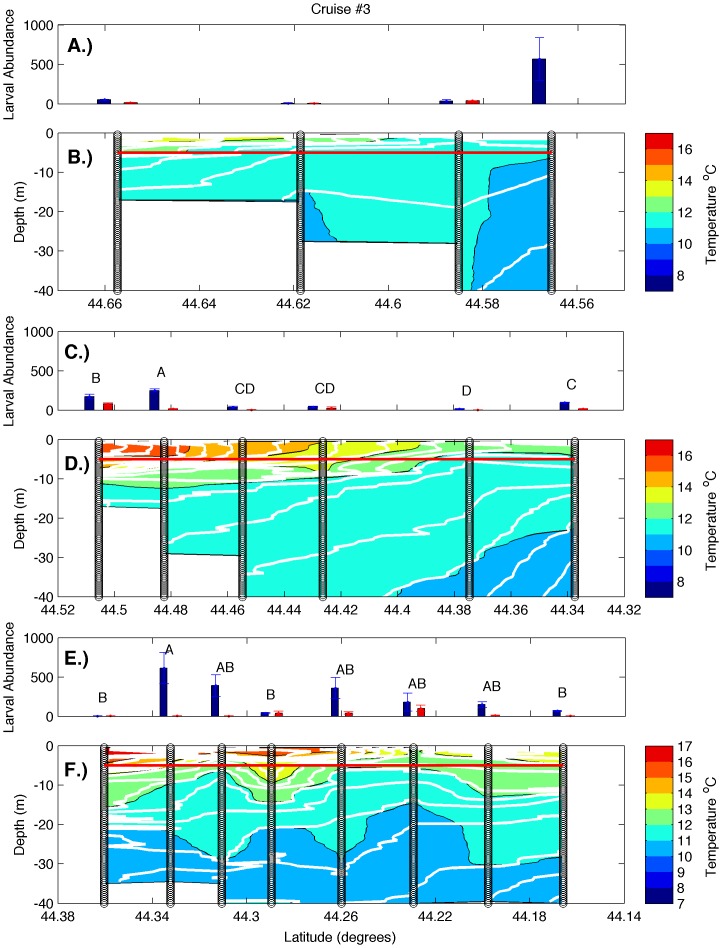
Larval abundance and water column structure on August 3–6, 2010. Panels represent larval abundance and water column structure, respectively, for transects 1 (A, B), 2 (C, D), and 3 (E, F). Larval abundance is presented as larvae m^−3^ at 5 m (blue bars) and 0.25 m (red bars). Error bars represent one standard error. The red line indicates the depth at which 5 m samples were collected. Variation in larval density among stations was significant on transects 2 and 3 (one-way ANOVA, p<0.0001 and p<0.01, respectively), and letters indicate 5 m stations that did not differ significantly from one another (Tukey-Kramer post-hoc test, p>0.05). Vertical black lines represent the locations of CTD and larval sampling stations, color gradations represent water temperatures, and the white contour lines represent isopycnals.

**Figure 5:**
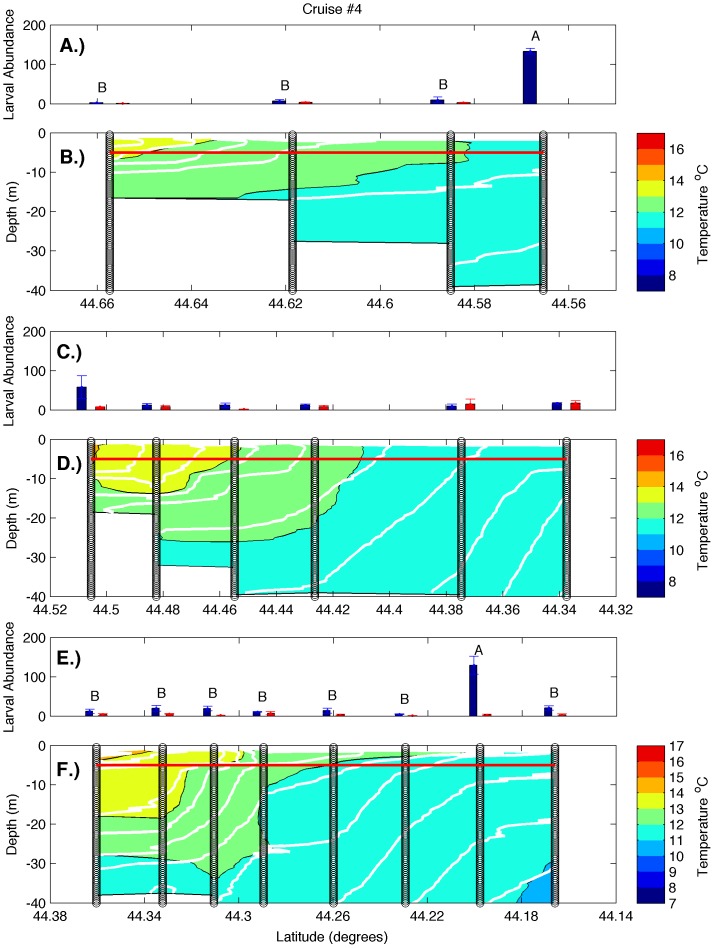
Larval abundance and water column structure on August 24–26, 2010. Panels represent larval abundance and water column structure, respectively, for transects 1 (A, B), 2 (C, D), and 3 (E, F). Larval abundance is presented as larvae m^−3^ at 5 m (blue bars) and 0.25 m (red bars). Error bars represent one standard error. The red line indicates the depth at which 5 m samples were collected. Variation in larval density among stations was significant on transects 1 and 3 (one-way ANOVA, p<0.0001 for both cases), and letters indicate 5 m stations that did not differ significantly from one another (Tukey-Kramer post-hoc test, p>0.05). Vertical black lines represent the locations of CTD and larval sampling stations, color gradations represent water temperatures, and the white contour lines represent isopycnals.

Examination of hydrographic transects during all cruises (Figures 2, 3, 4, 5) revealed a warm, strongly stratified inshore region and a vertically mixed offshore region that was dominated by the colder waters of the EMCC. Variation in salinity was minimal and followed a similar pattern of inshore stratification and offshore mixing (data not shown). Isopycnals (white lines in Figures 2, 3, 4, 5) closely mirror the locations of isotherms (colored contours in Figures 2, 3, 4, 5), indicating that temperature accounted for most of the density variation present. Consequently, the remainder of our analysis and discussion is restricted to temperature. Surface temperatures varied from 9°C in early June to 17°C in late August in the inshore region, and from 8°C to 12°C in the offshore region. These temperatures are consistent with the long-term climatology of sea surface temperature as revealed in AVHRR data (Figure 1), both in temporal and spatial patterns. Surface temperature and the across-shelf extent of the stratified inshore region increased to the southwest, away from the mouth of the Bay of Fundy and the source of the cold EMCC. Deeper temperatures at all stations were similar during any individual cruise but warmed over the summer. The vertical and horizontal structure of the inshore, stratified region also varied in the alongshelf direction. Transect 1 (i.e. the closest to mouth of the Bay of Fundy) was characterized by a relatively shallow plume of warm water (<5 m) that extended approximately 4 km offshore. The offshore region was occupied by EMCC temperature water [28]. Transects 2 and 3 were characterized by progressively greater vertical stratification that extended to greater depths (>10 m) and further distances offshore (>12 km). Frequently, the vertical stratification extended to the most offshore station of transects 2 and 3, indicating that temperature (and larval abundance) stations reached only the inshore edge of the EMCC. These vertical transects are consistent with the sea surface temperature climatologies for June-August (Figure 1) that show that the cold surface waters of the EMCC tend to be bounded inshore by the 75 m isobath (white line in Figure 1) which moves offshore as the continental shelf expands southeastward.

### Moored Observations

**Figure 6:**
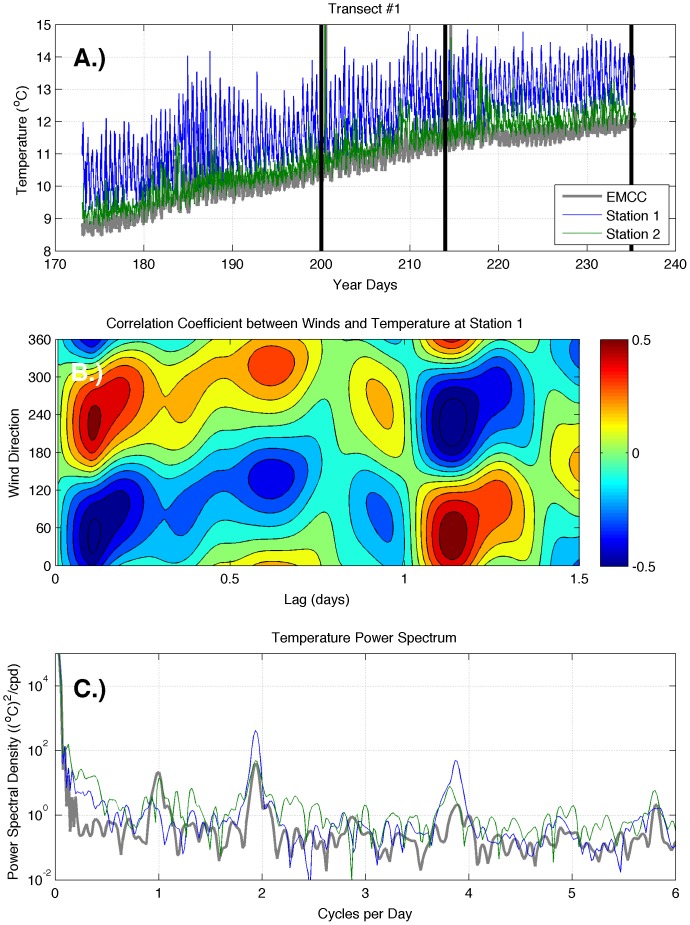
Temperature time series data (A) and analysis (B, C) for transect 1. The two analysis panels present wind-temperature correlations (B), and the power spectra of surface temperature (C). Station numbers begin near the coast and increase offshore. Vertical black lines indicate times of cruises. Note that wind direction refers to the direction that wind is blowing *from*.

Examination of all moored temperature measurements reveals that transect 1 was characterized by strong spatial and temporal variation in temperature. The time series of temperature at 5 m on transect 1 (Figure 6a) showed a strong across-shelf gradient that is consistent with the across-shelf vertical cross-sections. Station 1 was considerably warmer than stations 2 or 3. Station 3 of transect 1 was located near the cold core of the EMCC (Figure 1) and measured temperatures that typically characterize the EMCC [28]; consequently, we have used this station as a proxy for temperature values and variation in the EMCC during this study. There was much more variation at stations 1 and 2 than station 3 (EMCC). Frequently, the temperatures at stations 1 and 2 decreased to values of the EMCC. Power spectra of the temperature time series reveal a strong diurnal signal at stations 2 and 3, as well as a strong tidal signal at all three stations at both the M2 and M4 tidal frequencies (Figure 6c). There was also significant variance at lower frequencies (<0.02 cpd) at all stations and higher frequencies (0.2–0.5 cpd) at stations 1 and 2. To determine the relationship between winds and temperature fluctuations, we compared the 5 m temperature at the inshore mooring (station 1) of each transect with the components of wind oriented in all directions. There was a significant negative correlation (r<−0.4, lag = 0.1 days) between northeasterly winds (∼40–60°N) and 5 m temperature at station 1 (Figure 6b), as well as a positive correlation (r>0.5) at a lag of approximately 1.1 days. Note that a positive (negative) correlation at a given wind direction will result in an equal amplitude negative (positive) correlation for a wind direction that differs by 180°; consequently we describe here only those correlations with winds from 0–180°N.

**Figure 7:**
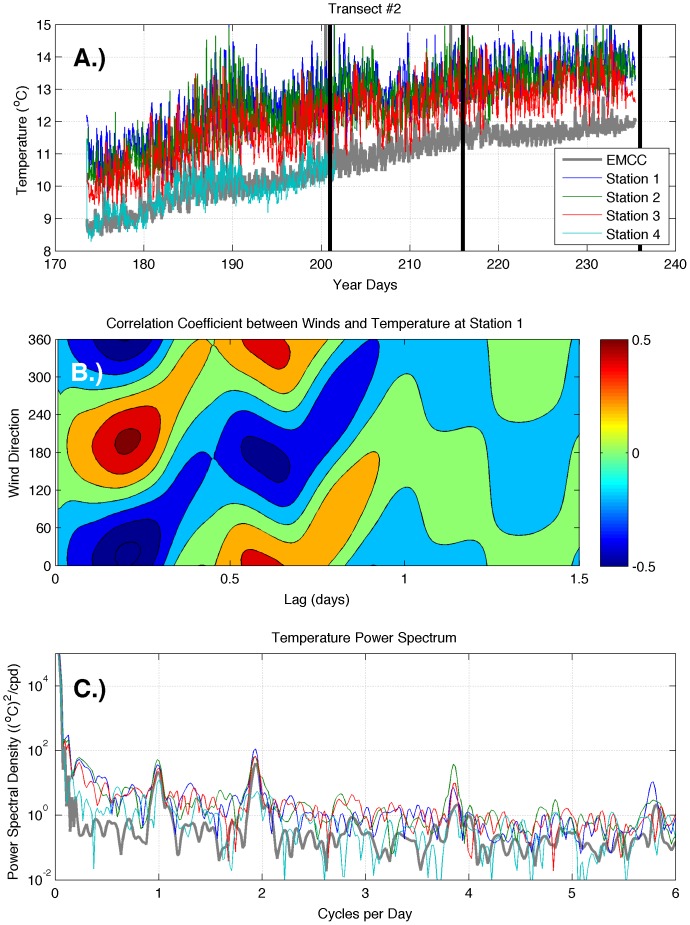
Temperature time series data (A) and analysis (B, C) for transect 2. The two analysis panels present wind-temperature correlations (B), and the power spectra of surface temperature (C). Station numbers begin near the coast and increase offshore. Vertical black lines indicate times of cruises. Note that wind direction refers to the direction that wind is blowing *from*.

Transect 2 was also characterized by strong spatial and temporal variation in temperature. Temperatures at station 4 of transect 2 (light blue line in Figure 7a) were similar to the EMCC (gray line in Figure 7a). Although stations 1–3 on transect 2 were generally warmer than station 4 (and the EMCC), there were periods of time when the temperatures at these inshore stations decreased to those of the EMCC. Temperatures at all stations varied at tidal (M2 and M4) and diurnal frequencies (Figure 7c). The inshore temperatures (stations 1–3) also varied at higher frequencies (0.2–0.5 cpd). The relationship between temperature and winds at transect 2 was similar to transect 1. Northerly winds (∼10–20°N) showed a negative correlation (r<−0.5, lag = 0.2 days) with temperature at station 1 (Figure 7b). And again, there was a strong but delayed positive correlation (r>0.3, lag = 0.6 days) between northerly winds and temperature.

**Figure 8:**
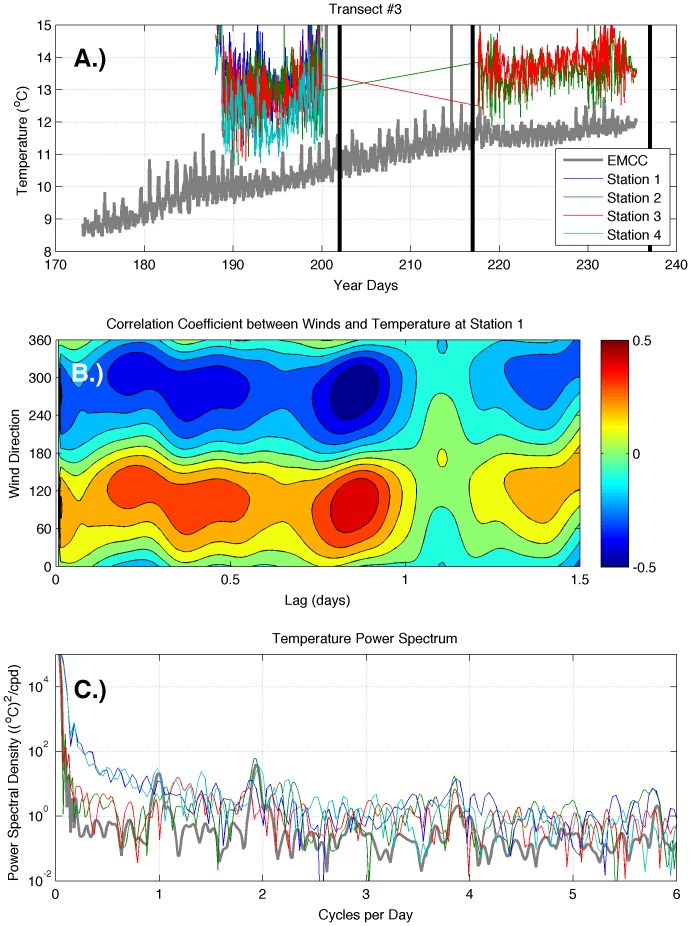
Temperature time series data (A) and analysis (B, C) for transect 3. The two analysis panels present wind-temperature correlations (B), and the power spectra of surface temperature (C). Station numbers begin near the coast and increase offshore. Vertical black lines indicate times of cruises. Note that wind direction refers to the direction that wind is blowing *from*.

Inshore temperatures on transect 3 (Figure 8a) rarely decreased to EMCC values. Although there was strong variability at all stations, temperature signals were much less evident at tidal frequencies along transect 3 (Figure 8c). Examination of the correlation between winds and temperature at station 1 of transect 3 (Figure 8b) reveals a very different relationship between winds and water temperatures than the two upshelf transects. There is a strong, positive correlation (r>0.5) between easterly winds (∼90–100°N) and temperature at station 1.

### Drifters

**Figure 9:**
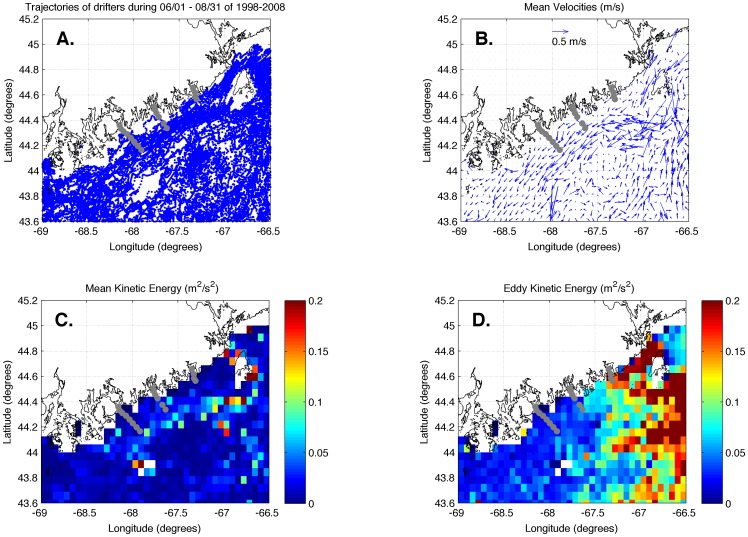
Trajectories (A) and analysis (B, C, D) of surface and drogued drifter data. The three analysis panels present mean velocities (B), mean kinetic energy (C), and eddy kinetic energy (D) calculated from drifters entering the study region from June 1 – August 31 of 1998–2009. Gray circles indicate CTD and larval sampling stations.

The trajectories of drifters deployed in the region provided evidence of strong variation in horizontal exchange of surface waters. A plot of the trajectories of all drifters released during the summers of 1998–2009 is shown in Figure 9a. While a number of the drifters became entrained into the southwestward-flowing EMCC, few if any were advected onshore by instabilities [14], wind-induced events [11], [40], or tidal currents [13]. Examination of the mean velocities calculated from the drifters (Figure 9b) and MKE (Figure 9c) show that the downstream speeds of the EMCC were strong (∼0.4 m s^−1^) within the region occupied by transects 2 and 3, but the majority of the EKE (Figure 9d), which is mostly associated with tidal velocities, was much greater at, or northeast of, transect 1.

### Larval Abundance

There was strong temporal and spatial variation in larval abundance during this study. On transect 1, larval bivalve densities varied from <50 m^−3^ to >500 m^−3^. During all cruises, the density of larvae in the 5 m samples was generally an order of magnitude greater than in the 0.25 m samples (Figures 2, 3, 4, 5). Actual counts at 0.25 m were often too low to support statistical analysis, so formal analysis is limited to those found at the 5 m depth. During the first two cruises, bivalve larvae were relatively rare at 5 m at all stations (Figures 2 & 3; note that y-axis scales vary among figures), consistent with expectations for these early season sampling dates (little spawning should have occurred in the colder waters upshelf of the study region). Abundance was higher on the subsequent two cruises, and in both cases, the outermost station was characterized by higher larval density than the other stations (Figures 4 & 5). However, this trend was only significant on cruise 4 (one-way ANOVA and subsequent post-hoc tests). Transect 2 was characterized by similar magnitudes and temporal fluctuations in larval abundance, but the pattern of spatial variation was quite different. During all cruises, bivalve larval abundance tended to be higher at inshore stations and was significantly so for three out of the four cruises (Figures 2, 3, 4, 5; one-way ANOVAs, see post-hoc results in the figures for station-specific details). In contrast to the other two transects, larval abundance on transect 3 never exhibited a clear onshore/offshore pattern. Bivalve densities on cruises 1 and 2 did not vary significantly among stations (Figures 2 and 3, one-way ANOVA). While there was significant variation among stations during cruises 3 and 4, peak abundance occurred at intermediate stations (Figs. 4 & 5, one-way ANOVA and subsequent post-hoc tests).

## Discussion

### Physical Flow Field

**Figure 10:**
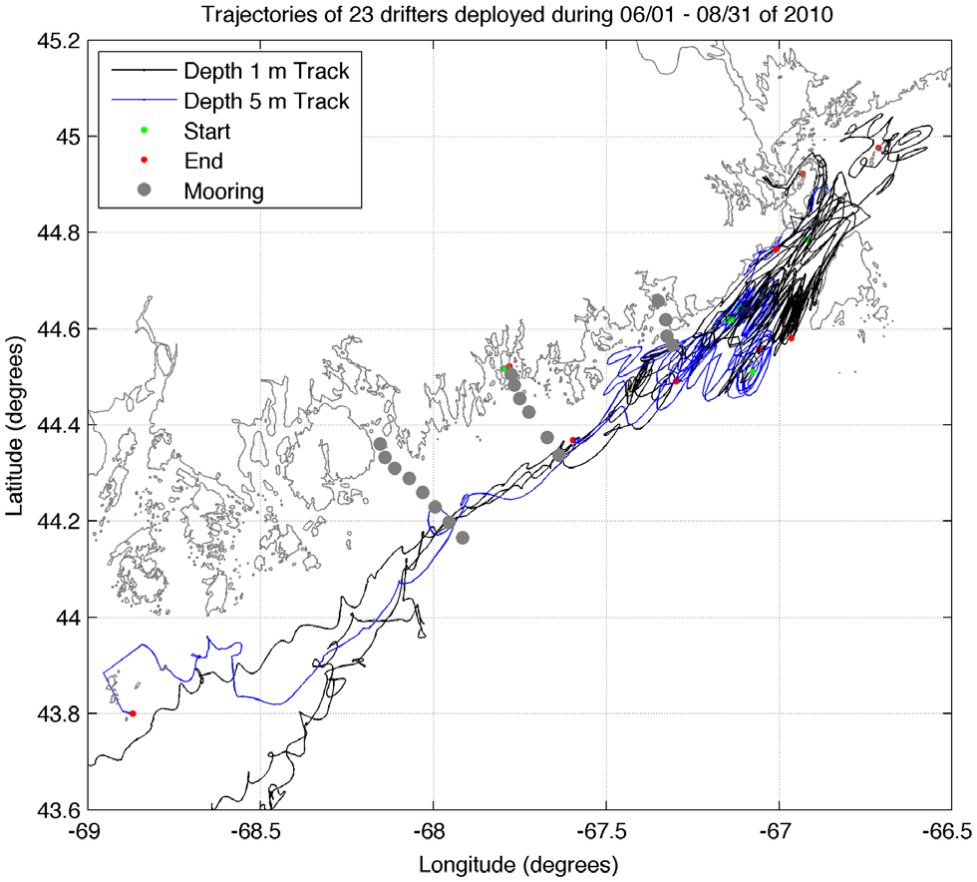
Trajectories of surface (blue) and drogued (black) drifters deployed June 1 – August 31, 2010. Green circles indicate starting point of each trajectory. Red circles indicate end of each trajectory. Gray circles indicate CTD and larval sampling stations.

The observed spatial variation in temperature is consistent with a flow field that is governed by interaction with the bottom topography of the region and vertical stratification from river run-off, as well as wind- and tidal-driven mixing with the EMCC [29]. Both observations [28] and numerical models of the region [41] show that the strongest currents in the region occur within the EMCC. However, our study area is centered on a continental shelf that tends to protect the inshore region from the strong currents and associated mixing of the EMCC [42], [43]. Moving southwestward along the coastline, away from the mouth of the Bay of Fundy, the core of the EMCC occurs further from the coastline and is replaced by a stratified inshore environment. Some authors have attributed this vertical stratification to river discharge emanating from the bays and estuaries along the coast [44], while others have postulated that the gradual separation of the EMCC from the coast is due to a combination of fresher and warmer waters inshore, topographic steering, and interaction with slope waters in the Jordan Basin [29]. The trajectories of drifters released in the immediate area both before (Figure 9a) and during (Figure 10) this study support the presence of reduced across-shelf exchange between inshore and offshore regions. Our study shows that the drifters rarely enter the inshore regions of transects 2 and 3; however they frequently occur close to shore at transect 1 and in the portion of the EMCC northeast of transect 1. This is consistent with a study of drifter tracks in the Gulf of Maine [33], which found that all drifters that entered the EMCC continued alongshelf.

The strong currents of the EMCC and its associated mixing are responsible for the drastically different vertical structure of the water column at the different transects along the Maine coast [28]. Due to its proximity to the mouth of the Bay of Fundy, transect 1 (Machias Bay) experiences greater tidal-induced vertical and horizontal exchange (Figure 9d). The greater mixing results in less stratification in both the vertical and horizontal directions (top panels of Figures 2, 3, 4, 5) along transect 1. Transect 3 (Frenchman Bay) exhibits the greatest horizontal gradients and vertical stratification and weakest EKE (Figure 9d). Examination of the tracks of drifters released during this study (Figure 10) illustrates the strong geographic variation of EKE and its effect on alongshelf transport. Near the mouth of the Bay of Fundy, the flow is restricted by the Maine coastline and Grand Manan Island within the Grand Manan Channel. Here, the drifters tend to move back and forth with the tidal currents. The strong velocities and, therefore, horizontal shear result in more horizontal exchange, less stratification, and greater across-shelf transport between the inshore environment and the EMCC. Once water leaves this region, the flow field expands and is no longer dominated by tides. Instead, buoyancy-generated, topographically-constrained geostrophic currents result in immediate downstream transport of waters and material within the EMCC. This downstream transport is true for both surface drifters (blue lines in Figure 10) and those drogued at 5 m (black lines in Figure 10).

**Figure 11:**
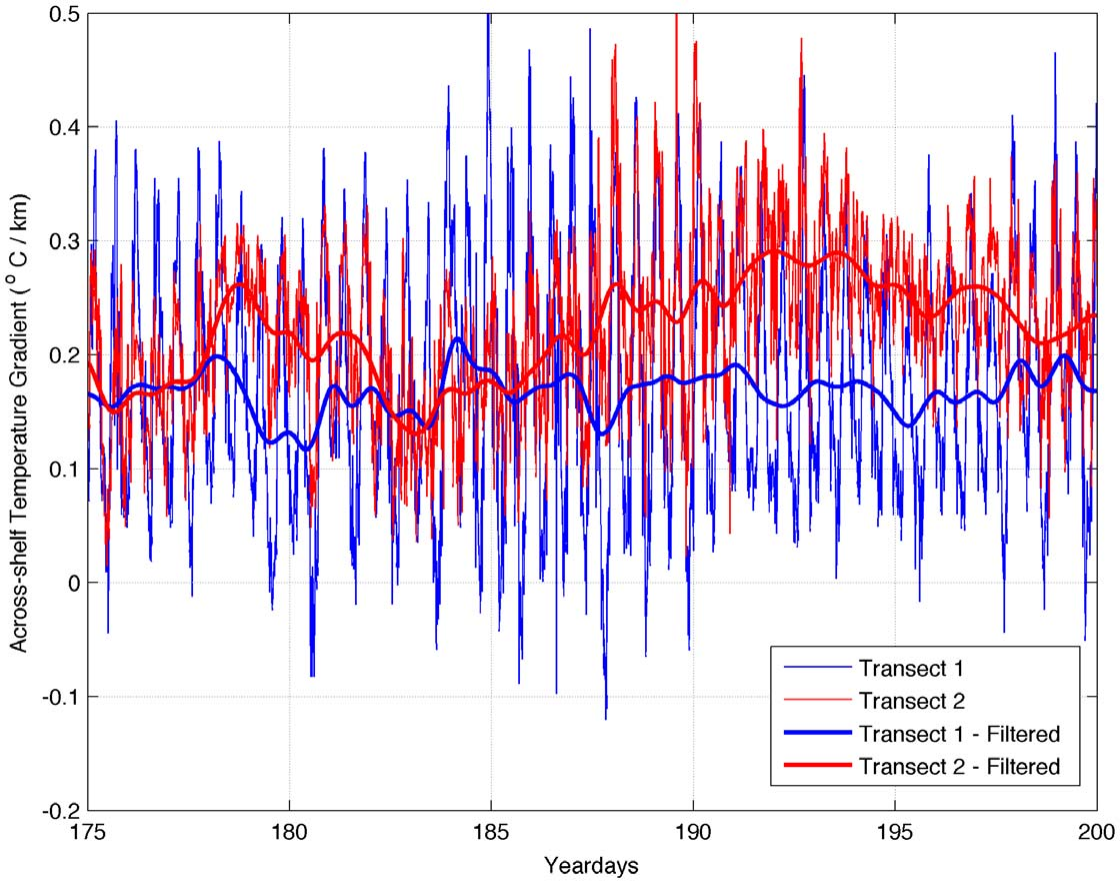
Across-shelf gradient of temperature (°C/km) along transects 1 (blue line) and 2 (red line). Thin lines are unfiltered temperature. Thick lines have been filtered with a Lanczos filter with a 1/36 hr^−1^ cut off frequency.

Although the four cruises produced only a few snapshots of the across-shelf and vertical structure, comparison of the across-shelf temperature gradients at transects 1 and 2 calculated from the moored temperatures (Figure 11) confirms that transect 2 (Pleasant Bay) was typically characterized by greater horizontal gradients than transect 1 (Machias Bay). Examination of the unfiltered data revealed a strong tidal signal at both transects. Transect 1 was characterized by stronger across-shelf temperature gradients during low tide and weaker across-shelf temperature gradients during high tide than transect 2, but the mean temperature gradient value at transect 1 was significantly lower than that at transect 2 (0.17 vs. 0.21°C km^−1^). Although the coarseness of this gradient calculation precludes the identification of fronts in which larvae would accumulate [23], the difference in the calculated large-scale gradients is consistent with the closer proximity of the well-mixed EMCC to the coast at transect 1.

The observed spatial and temporal variation in temperature along the transects is consistent with a wind-driven flow field affected by interactions with an EMCC that varies in its distance from shore. The frequent decrease of temperatures at stations 1 and 2 along transect 1 (Machias Bay) to values of the EMCC (Figure 6a) suggests that the inshore edge of the EMCC (which is characterized by strong horizontal gradients) episodically passed by stations 1 and 2, but station 3 remained within the core of the EMCC. The strong diurnal temperature signals at stations 2 and 3 suggest daily solar heating (Figure 6c). There was also significant variance at frequencies associated with seasonal variation of surface heating (<0.02 cpd) at all stations and wind-forced transport and exchange (0.2–0.5 cpd) at stations 1 and 2 as the EMCC entered the inshore region. Winds affected temperature fluctuations along transect 1 through two different physical mechanisms. The direction of the highest correlated winds (∼40–60°N) and the absence of a significant time lag between the winds and temperature (Figure 6b) indicate that the variation in temperature was not due to wind-induced upwelling of cold waters [12]. Because the downshelf orientation of the coastline at transect 1 is approximately 225°N, northeasterly winds (∼40–60°N) would result in downwelling, not upwelling. However, northeasterly winds did result in advection of the cold EMCC onshore due to Ekman transport, which immediately decreased the surface temperature near the coast. Southwesterly (∼220–240°N) winds resulted in offshore movement of the EMCC and warming of the inshore waters. The lagged, positive (negative) correlation between northeasterly (southwesterly) winds and temperature suggest that, at longer time scales, winds affect temperatures of the coastal waters through the traditional coastal upwelling/downwelling mechanism [12], [45]. The time lag associated with northeasterly and southwesterly winds is consistent with the extended time period required for Ekman spin up for downwelling and upwelling (respectively) [12], [46].

The spatial and temporal variation in temperature along transect 2 was also driven by tides and wind-forced advection of the EMCC. The similarity between temperatures at station 4 of transect 2 (light blue line in Figure 7a) and the EMCC (gray line in Figure 7a) suggests that station 4 was consistently located in the EMCC. The decreases in temperatures at stations 1–3 indicate that the EMCC migrated onshore. Again, the winds affected surface temperature at different time scales in a similar manner to transect 1. The immediate negative (positive) correlation between northerly (southerly) winds and temperatures at station 1 (Figure 7b) suggests wind-driven onshore (offshore) advection of the EMCC. The lagged positive (negative) correlation between northerly (southerly) winds and temperatures is again consistent with downwelling (upwelling) of coastal waters.

The inshore region at transect 3 was affected by tides and wind-driven flow of stratified inshore waters, but not the presence of the EMCC. Inshore temperatures at transect 3 (Figure 8a) rarely decreased to EMCC values, indicating that the EMCC most likely did not migrate onshore past stations 1–4. Although there was strong variability at all stations, the lack of resolved variation in temperature at tidal frequencies along transect 3 (Figure 8c) indicates that the region was not subjected to strong tidal-induced variation from the across-shelf movement of colder offshore waters and warmer inshore waters. The lack of a diurnal signal suggests that solar heating played less of a role than other mechanisms in determining temperatures along the transect. The strong, positive (negative) correlation between easterly (westerly) winds and temperature is consistent with large-scale upwelling (downwelling) of deeper waters in a frictional environment, in which the wind direction of greatest influence on surface transport is not directly parallel to the coast but is instead oriented at an angle to the coastline [40], [47]. Examination of bottom topography (Figure 1) reveals that transect 3 was located within a drowned river valley and station 1 is in water whose depth is greater than 75 m. There was no evidence of the advection of the EMCC into the region from the temperature signal or the relationship between winds and temperature, as at transects 1 and 2.

### Larval Abundance

In order to improve sampling synopticity and examine the effects of possible vertical migration, all of our larval samples were collected at fixed depths of 0.25 and 5 m. Samples from stations within the EMCC were collected from a vertically well-mixed water column, while samples from intermediate stations were in more stratified water, but generally above the pycnocline if one was present (Figures 2, 3, 4, 5). While we feel that vertical migration by larvae is unlikely to have affected dispersal trajectories in the well-mixed EMCC, vertical migration could have played a role in establishing the distributions reported for the inshore waters (i.e., for larvae produced in the inshore that were on an out-bound trajectory). Since we sampled at 0.25 and 5 m depths and mussel larvae (which constituted the vast bulk of our larval samples) have rarely been reported deeper than 7–8 m [48], [49], the relevant un-sampled layer appears to be quite small. Larval abundance was overall much lower in corresponding 0.25 m samples (red bars in Figures 2, 3, 4, 5) than at 5 m (blue bars in Figures 2, 3, 4, 5). Even at locations where the pycnocline was at a depth <5 m (and vertical migration of larvae would have the greatest effect on horizontal transport), the 5 m stations were characterized by much higher larval density. Consequently, we have little evidence that vertical migration occurs in this system. While we fully appreciate the potential role of vertical migration in altering larval dispersal trajectories in some regions, we have little reason to suspect that vertical migration played a major role in generating the large-scale patterns reported here.

The mean horizontal velocities within even the inshore region (∼0.1 m/s, see Figure 9b) were significantly greater than the swimming velocities of the largest bivalve larvae (e.g., 0.008 m/s for late stage oyster larvae – [50]; 0.004 m/s for late stage mussel larvae – [51]). Since vertical migration of larvae most likely did not dramatically alter dispersal trajectories (see discussion above), the horizontal larval distribution was probably largely determined by the flow field. However, the greater stratification within the inshore region in both the horizontal and vertical dimensions has implications for the horizontal exchange and transport of passive material. The variation in spatial patterns of larval abundance along the different transects can be explained by variations in larval sources as well as differences in the offshore distance of the EMCC, horizontal structure, and EKE, which all result in different amounts of across-shelf exchange.

The lack of resolved variation in larval abundance among stations in transect 1 during three out of the four cruises (top panels of Figures 2, 3, 4) is consistent with greater EKE (Figure 1) and subsequent horizontal exchange that would lead to less spatial variation in both temperature (Figure 11) and passive material suspended within the water column. However, the higher larval density at station 4 than the other stations during cruise 4 (Figure 5) suggests that the offshore EMCC can contain bivalve larvae that originated to the northeast of the study region.

The stronger horizontal variation and weaker EKE along transects 2 and 3 result in less across-shelf exchange along those transects and greater variation in larval abundance among stations. Larval densities were significantly greater at the inshore end of transect 2 on three of the four cruises. Higher bivalve larval abundance at inshore stations suggests that larvae were derived from a different source than those present on transect 1, with that source probably located in the inshore region between transects 1 and 2. The aggregation of bivalve larvae present on the offshore end of transect 1 during cruise 4 was not detected on the offshore end of transect 2, which is consistent with dilution due to exchange during alongshelf transport within the EMCC. The presence of station effects on transect 2 suggests that little offshore transport of larvae occurred during these cruises, although the similarity of larval densities among closely adjacent stations supports exchange on smaller scales.

Transect 3 exhibited the greatest horizontal variation and weakest EKE. In two of the four cruises, larval densities varied among stations, and this pattern is consistent with little exchange. During all four cruises, there was no pattern in larval density that would suggest a nearby source at either end of the transect. The peak bivalve larval abundance at the intermediate stations often appeared to be associated with a boundary between water masses (Figure 5). The elevated abundance could represent accumulation of the larvae in a convergence zone; however, this occurrence would require vertical swimming behavior of the larvae.

In the absence of wind-driven inshore migrations of the EMCC, our results suggest that the interface between the EMCC and inshore waters may constitute a bidirectional ecological barrier to larval dispersal. Indirect evidence supports this hypothesis for larvae originating northeast of the study region. The range boundary of the northern blue mussel, *Mytilus trossulus*, corresponds closely with the EMCC/inshore water interface [22]. Because all substantial larval source populations for this species are northeast of the study area, the lack of exchange of larvae across the EMCC/inshore interface may limit the geographic extent of this species. Similarly, settlement of green sea urchins (*Strongylocentrotus droebachiensis*) has historically been lower in this region (1996–1998) than in inshore regions further to the southwest [20]. As urchin populations inshore of the EMCC were subsequently eradicated by harvesting (from 2001–2008), and only upshelf larval source populations remained, recruitment to the fishery in this region dropped dramatically [52]. No similar decrease in recruitment was observed along the shore within the Grand Manan channel, where the EMCC can abut the shore ([52] and Figure 1). This comparison suggests that historically, locally-produced sea urchin larvae were retained inshore of the EMCC and some degree of self-seeding occurred. Since the eradication of that local larval source, recruitment from upshelf populations has been very limited. This example may be complicated by regional differences in post-settlement survival due to variation in predation pressure mediated by habitat modification induced by the removal of urchins [53]. While the scenario is inevitably even more complicated for other taxa with larval sources both upshelf and inshore of the EMCC, some evidence of a similar ecological barrier exists. We have consistently observed recruitment of mussels to settlement plates deployed at the inshore ends of transects 2 and 3 at times when no larvae were present in the EMCC, but inshore larval abundance was high (unpublished data). These settlement events may well reflect local retention of larvae of the southern blue mussel, *M. edulis*, which failed to be transported outward into the EMCC. Finally, the entire region inshore of the EMCC has previously been recognized as a low recruitment zone for lobsters (*Homarus americanus*) [53]. Southwest of the EMCC, where the Western Maine Coastal Current appears to exchange waters with the inshore region [30], lobster recruitment is enhanced [53]. The precise larval source/sink relationships are not clear in this species (likely source populations extend north into the Canadian Maritime Provinces and south of Cape Cod), and recruitment patterns may be complicated by temperature dependent onshore/offshore post-settlement mortality patterns [54], more sophisticated behavior by the strongly-swimming larvae of this species, and longer-term trends in spatial variation in recruitment. Nevertheless, the reduction in recruitment in a region with only limited access to upshelf larvae is consistent with upshelf enhancement for other regions.

While this study examined only four isolated snapshots of larval abundance, the strong relationship between winds and temperature at all stations (middle panels of Figures 2, 3, 4, 5) suggests that possible episodic coastal delivery of larvae present in the EMCC may be related to wind-induced transport. Decreased temperatures indicating onshore movement of the EMCC were correlated with alongshelf (but not upwelling) winds, and competent larvae that enter the study region via the EMCC could settle during inshore migrations of the EMCC. Our understanding of wind effects combined with better data on temporal patterns of larval availability would allow us to predict and even quantify these events, providing resource managers and scientists with a tool to predict future settlement events. Our existing data do suggest that such wind-driven onshore migrations might be more common along transect 1 but rarer in the vicinity of transects 2 and 3 (Figures 7 and 8), where the presence of the interface between inshore waters and the EMCC results in only limited onshore migration. The wind events responsible for onshore migration of larvae from the EMCC at any location within our study are episodic. During our three month study, 6.0% of winds whose speeds exceeded 2 m/s were northeasterly (40–60°N), which would result in onshore migration of the EMCC along transect 1, creating 10 wind events that exceeded 4 hours and could transport larvae in the EMCC into the inshore areas. These wind events were more common than those that would result in EMCC migration towards shore along transects 2 (∼10–20°N) and 3 (90–100°N), which occurred 2.2% and 2.8% of the time, respectively. Unless the timing of the infrequent wind-driven exchange coincides with peaks in offshore larval availability, few larvae are likely to be transported inshore by this mechanism in these regions where the EMCC diverges from the coast.

## References

[1] Cowen RK, Gawarkiewicz G, Pineda J, Thorrold S, Werner F (2002) Population connectivity in marine systems. Report of a Workshop to Develop Science Recommendations for the National Science Foundation

[2] Wing SR, Largier JL, Botsford LW, Quinn JF (1995) Settlement and Transport of Benthic Invertebrates in an Intermittent Upwelling Region. Limnol Oceanogr 40: 316–329.

[3] Epifanio CE, Garvine RW (2001) Larval transport on the Atlantic continental shelf of North America: a review. Estuar Coast Shelf Sci 52: 51–77.

[4] Shanks AL, Brink L (2005) Upwelling, downwelling, and cross-shelf transport of bivalve larvae: test of a hypothesis. Mar Ecol Prog Ser 302: 1–12.

[5] Morgan SG, Fisher JL (2010) Larval behavior regulates inshore retention and offshore migration in an upwelling shadow and along the open coast. Mar Ecol Prog Ser 404: 109–126.

[6] Criales MM, Browder JA, Mooers CNK, Robblee MB, Cardenas H (2007) Cross-shelf transport of pink shrimp larvae: interactions of tidal currents, larval vertical migrations and internal tides. Mar Ecol Prog Ser 345: 167–184.

[7] Chicharo LMZ, Chicharo MA (2000) Estimation of the life history parameters of *Mytilus galloprovincialis (*Lamarck) larvae in a coastal lagoon (Ria Formosa - south Portugal). J Exp Mar Bio Ecol 243: 81–94.

[8] Shanks AL (2006) Mechanisms of cross-shelf transport of crab megalopae inferred from a time series of daily abundance. Mar Biol 148: 1383–1398.

[9] Cudaback CN, McPhee-Shaw E (2009) Diurnal-period internal waves near point conception, California. Est Coast Shelf Sci 83: 349–359.

[10] Epifanio CE, Tilburg CE (2008) Transport of larval forms near large estuaries of the Middle Atlantic Bight: A wet and windy journey. J Mar Res 66: 723–749.

[11] Fong DA, Geyer WR (2001) Response of a river plume during an upwelling favorable wind event. J Geophysical Res 106: 1067–1084.

[12] Austin JA, Lentz SJ (2002) The inner shelf response to wind-driven upwelling and downwelling. J Phys Oceanogr 32: 2171–2193.

[13] Sanders TM, Garvine RW (2001) Fresh water delivery to the continental shelf and subsequent mixing: an observational study. J Geophys Res 106: 27087–27101.

[14] Mork M (1981) Circulation phenomena and frontal dynamics of the Norwegian coastal current. Phil Trans Royal Soc London A302: 635–647.

[15] Jones MB, Epifanio CE (1995) Settlement of brachyuran megalopae in Delaware Bay: a time series analysis. Mar Ecol Prog Ser 125: 67–76.

[16] Bishop TD, Miller HL, Walker RL, Hurley DH, Menken T, et al (2010) Blue crab (*Callinectes sapidus* Rathbun, 1896) settlement at three Georgia (USA) estuarine sites. Est Coasts 33: 688–698.

[17] Ogburn MB, Diaz H, Forward RB Jr (2009) Mechanisms regulating estuarine ingress of blue crab *Callinectes sapidus* megalopae. Mar Eco Prog Ser 389: 181–192.

[18] Garvine RW, Epifanio CE, Epifanio CC, Wong KC (1997) Transport and recruitment of blue crab larvae: a model with advection and mortality. Est Coast Shelf Sci 45: 99–111.

[19] Tilburg CE, Whitney MM, Reager JT (2005) The physics of blue crab larval recruitment in Delaware Bay: A model study. J Mar Res 63: 471–495.

[20] McNaught DC, Steneck RS (1998) Settlement and survival of the green sea urchin in Maine: Effects of algal habitat. Final report to the Maine Department of Marine Resources

[21] Xue H, Incze L, Xu D, Wolff N, Pettigrew N (2008) Connectivity of lobster populations in the coastal Gulf of Maine Part I: Circulation and larval transport potential. Ecol Modeling 210: 193–211.

[22] Hayhurst S, Rawson PD (2009) Species-specific variation in larval survival and patterns of distribution for the blue mussels *Mytilus eduli* and *Mytilus trossulus* in the Gulf of Maine. J Molluscan Studies 75: 215–222.

[23] Woodson CB, McManus MA, Tyburczy JA, Barth JA, Washburn L, et al (2012) Coastal fronts set recruitment and connectivity patterns across multiple taxa. Limnol Oceanogr 57: 582–596.

[24] Xue H, Chai F, Pettigrew NR (2000) A model study of seasonal circulation in the Gulf of Maine. J Physical Oceanogr 30: 1111–1135.

[25] Mupparapu P, Brown WS (2002) Role of convection in winter mixed layer formation in the Gulf of Maine, February 1987. J Geophys Res 107: 3229–3245.

[26] Hetland R, Signell RP (2005) Modeling coastal current transport in the Gulf of Maine. Deep Sea Res Part II 52: 2430–2449.

[27] Pettigrew NR, Townsend DW, Xue H, Wallings JP, Brickley PJ, et al (1998) Observations of the Eastern Maine Coastal Current and its offshore extensions. J Geophys Res 103: 30623–30639.

[28] Pettigrew NR, Churchill JH, Janzen CD, Mangum L, Signell RP, et al (2005) The kinematic and hydrographic structure of the Gulf of Maine Coastal Current. Deep Sea Res Part II 52: 2369–2391.

[29] Brooks DA (1985) Vernal circulation in the Gulf of Maine. J Geophysical Res 90: 4687–4705.

[30] Geyer WR, Signell RP, Fong DA, Wang J, Anderson DM, et al (2004) The freshwater transport and dynamics of the western Maine coastal current. Cont Shelf Res 24: 1339–1357.

[31] Brown WS, Irish JD (1992) The annual evolution of geostrophic flow in the Gulf of Maine: 1986–1987. J Phys Oceanogr 22: 445–473.

[32] Davis R (1985) Drifter observations of coastal surface currents during code: the method and descriptive view. J Geophys Res 90: 4741–4755.

[33] Manning JP, McGillicuddy DJ, Pettigrew NR, Churchill JH, Incze LS (2009) Drifter observations of the Gulf of Maine Coastal Current. Cont Shelf Res 29: 835–845. 28966432 10.1016/j.csr.2008.12.008 PMC5617362

[34] Garraffo ZD, Mariano AJ, Griffa A, Veneziani C, Chassignet EP (2001) Lagrangian data in a high-resolution numerical simulation of the North Atlantic: I. Comparison with in situ drifter data. J Mar Syst 29: 157–176.

[35] Emery WJ, Thomson RE (2001) Data Analysis Methods in Physical Oceanography, 2^nd^ edition. Amsterdam: Elsevier Science. 638 p.

[36] Bretherton CS, Widmann M, Dyminikov VP, Wallace JM, Blade I (1999) The effective number of spatial degrees of freedom of a time-varying field. J Climate 12: 1990–2009.

[37] Lutz RA, Kennish MJ (1992) Ecology and morphology of larval and early post-larval mussels. In: Gosling, E, editor. The mussel *Mytilus:* ecology, physiology, genetics and culture. Amsterdam: Elsevier. pp. 53–86.

[38] Rawson PD, Secor CL, Hilbish TJ (1996) The effects of natural hybridization on the regulation of doubly uniparental mtDNA inheritance in blue mussels (*Mytilus* spp.). Genetics 144: 241–248. 8878689 10.1093/genetics/144.1.241 PMC1207497

[39] Forward RB, Tankersley RA (2001) Selective tidal-stream transport of marine animals. Oceanogr Mar Biol 39: 305–353.

[40] Tilburg CE (2003) Across-shelf transport on a continental shelf: Do across-shelf winds matter? J Physical Oceanogr 33: 2675–2688.

[41] Lynch DR, Holboke MJ, Naimie CE (1997) The Maine coastal current: spring climatological circulation. Cont Shelf Res 17: 605–634.

[42] Beardsley RC, Chapman DC, Brink KH (1985) The Nantucket shoals flux experiment (NSFE79). Part I: A basic description of the current and temperature variability. J Phys Oceanogr 15: 713–748.

[43] Kelly KA, Chapman DC (1988) The response of stratified shelf and slope waters to steady offshore forcing. J Phys Oceanogr 18: 906–925.

[44] Bisagni JJ, Gifford DJ, Ruhsam CM (1996) The spatial and temporal distribution of the Maine Coastal Current during 1982. Cont Shelf Res 16: 1–24.

[45] Tilburg CE, Garvine RW (2003) Three-dimensional flow in a shallow coastal upwelling zone: alongshore convergence and divergence on the New Jersey shelf. J Physical Oceanogr 33: 2113–2125.

[46] Garvine RW (1991) Subtidal frequency estuary-shelf interaction: observations near Delaware Bay. J Geophys Res 96: 7049–7064.

[47] Garvine RW (1971) A simple model of coastal upwelling dynamics. J Phys Oceanogr 1: 169–179.

[48] Dobrestsov SV, Miron G (2001) Larval and post-larval vertical distribution of the mussel M*ytilus edulis* in the White Sea. Mar Ecol Prog Ser 218: 179–187.

[49] Freeman KR, Kenchington E, Macquarrie SP (2002) Comparative settlement depths of *Mytilus edulis* C. Linnaeus, 1758 and *M. trossulus* Gould, 1850: I. A mesocosm study,. J Shellfish Res 21: 59–65.

[50] Hidu H, Haskin HH (1978) Swimming speeds of oyster larvae *Crassostrea virginica* in different salinities and temperatures. Estuaries 1 4 252–255.

[51] Bayne BL (1963) Responses of *Mvtilus edulis* larvae to increases in hydrostatic pressure. Nature (Lond.) 198: 406–407.

[52] Anonymous (2010) Maine's Sea Urchin Survey. Report by the Department of Marine Resources, 2 pages

[53] Steneck RS, Wilson C (2001) Large-scale and long-term, spatial and temporal patterns in demography and landings of the American lobster, *Homarus americanus* . Mar Freshwater Res 52: 1303–1319.

[54] Annis ER (2005) Temperature effects on the vertical distribution of lobster postlarvae (*Homarus americanus*). Limnol Oceanogr 50: 1972–1982.

